# *Agastache* Species (Lamiaceae) as a Valuable Source of Volatile Compounds: GC–MS Profiling and Investigation of In Vitro Antibacterial and Cytotoxic Activities

**DOI:** 10.3390/ijms25105366

**Published:** 2024-05-14

**Authors:** Mihaela-Ancuța Nechita, Ioana-Ecaterina Pralea, Adrian-Bogdan Țigu, Cristina-Adela Iuga, Carmen Rodica Pop, Emese Gál, Rodica Vârban, Vlad-Ionuț Nechita, Ovidiu Oniga, Anca Toiu, Daniela Benedec, Daniela Hanganu, Ilioara Oniga

**Affiliations:** 1 Department of Pharmacognosy, Faculty of Pharmacy, “Iuliu Hațieganu” University of Medicine and Pharmacy, Ion Creangă Street 12, 400010 Cluj-Napoca, Romania; nechita_mihaela_ancuta@elearn.umfcluj.ro (M.-A.N.); atoiu@umfcluj.ro (A.T.); dhanganu@umfcluj.ro (D.H.); ioniga@umfcluj.ro (I.O.); 2 Department of Proteomics and Metabolomics, Research Center for Advanced Medicine–MedFuture, “Iuliu Hațieganu” University of Medicine and Pharmacy Cluj-Napoca, Louis Pasteur Street 4–6, 400349 Cluj-Napoca, Romania; pralea.ioana@umfcluj.ro (I.-E.P.); iugac@umfcluj.ro (C.-A.I.); 3 Department of Translational Medicine, Research Center for Advanced Medicine–MedFuture, “Iuliu Hațieganu” University of Medicine and Pharmacy Cluj-Napoca, Louis Pasteur Street 6, 400349 Cluj-Napoca, Romania; 4 Department of Pharmaceutical Analysis, Faculty of Pharmacy, “Iuliu Hațieganu” University of Medicine and Pharmacy, Louis Pasteur Street 6, 400349 Cluj-Napoca, Romania; 5 Department of Food Science, Faculty of Food Science and Technology, University of Agricultural Sciences and Veterinary Medicine of Cluj-Napoca, Calea Florești Street 64, 400509 Cluj-Napoca, Romania; carmen-rodica.pop@usamvcluj.ro; 6 Department of Chemistry and Chemical Engineering, Hungarian Line, Faculty of Chemistry and Chemical Engineering, Babeș-Bolyai University, Arany János Street 11, 400028 Cluj-Napoca, Romania; emese.gal@ubbcluj.ro; 7 Department of Crop Science, Faculty of Agriculture, University of Agricultural Sciences and Veterinary Medicine of Cluj-Napoca, Calea Mănăștur Street 3–5, 400372 Cluj-Napoca, Romania; rodica.varban@usamvcluj.ro; 8 Department of Medical Informatics and Biostatistics, Faculty of Medicine, “Iuliu Hațieganu” University of Medicine and Pharmacy, Louis Pasteur Street 6, 400349 Cluj-Napoca, Romania; nechita.vlad@umfcluj.ro; 9 Department of Pharmaceutical Chemistry, Faculty of Pharmacy, “Iuliu Hațieganu” University of Medicine and Pharmacy, Victor Babeș Street 41, 400010 Cluj-Napoca, Romania; ooniga@umfcluj.ro

**Keywords:** *Agastache* species, essential oils, antimicrobial, tumour cell lines, volatile compounds

## Abstract

Nowadays, there is an increasing interest in the study of medicinal and aromatic plants, due to their therapeutic properties that correlate with the presence of different active compounds. *Agastache* species (sp.) are aromatic plants that belong to the Lamiaceae family, originating from North America and East Asia. The present study aimed to evaluate the composition of essential oils (EOs) obtained from different Romanian cultivated *Agastache* sp. and to investigate their antibacterial and cytotoxic activities. The gas chromatography-mass spectrometry (GC–MS) screening revealed that menthone was the dominant constituent of *A. foeniculum* (31.58%), *A. rugosa* (39.60%) and *A. rugosa* ‘After Eight’ (39.76%) EOs, while estragole was the major constituent of *A. foeniculum* “Aromat de Buzău” (63.27%) and *A. mexicana* (41.66%) EOs. The investigation of the antiproliferative effect showed that *A. rugosa* and *A. foeniculum* “Aromat de Buzău” EOs had significant cytotoxic activity on MDA-MB-231 and HEPG2 tumour cell lines, with the most promising effect on the MDA-MB-231 breast cancer cell line for *A. foeniculum* “Aromat de Buzău” EO (IC_50_ = 203.70 ± 0.24 μg/mL). Regarding the antibacterial activity, *A. rugosa* EO was most active against *E. coli* (8.91 ± 3.27 μL/mL) and *S. aureus* (10.80 ± 0.00 μL/mL). To the best of our knowledge, this is the first report on the cytotoxic effect of *Agastache* sp. EOs on MDA-MB-231, HCT116 and HEPG2 tumour cell lines. The results of our study provide new and promising information for the subsequent in vivo study of the pharmacological properties of *Agastache* sp. essential oils.

## 1. Introduction

*Agastache* species (sp.) are aromatic plants from the Lamiaceae family that originate from North America and East Asia. Known under the common name of giant hyssop, they are used empirically in traditional medicine as a remedy for pain, hypertension, gastrointestinal disorders and infections [1,2,3,4]. They are also used as ornamental plants, spices and as an important source of essential oils (EOs) [1,2,3]. Some of the most studied sp. of the genus are *Agastache rugosa* (Fisch. & C.A.Mey.) Kuntze, *Agastache foeniculum* (Pursh) Kuntze and *Agastache mexicana* (Kunth) Lint & Epling [2,3,4].

*Agastache* sp. are less investigated compared to other medicinal plants from the Lamiaceae family, both from a phytochemical and therapeutic perspective. Studies evaluating the chemical composition showed that *Agastache* sp. extracts contain polyphenols, such as flavonoids (tilianin) [5] and phenolic acids (rosmarinic acid) [6]. The EOs from *Agastache* sp. contain terpenes (menthone, pulegone) [7] and phenylpropanoids (estragole) [8]. *Agastache* sp. extracts and EOs display a wide range of pharmacological properties, including antimicrobial [7,9], anti-inflammatory [10,11], anti-adipogenic, anti-atherosclerotic [12,13] and antiproliferative [7,11,14,15,16] properties. The in vitro antiproliferative effect was demonstrated against breast (MCF-7) [11,16], prostate (LNCaP) [14] and stomach (SGC-7901) [7,15] malignant cell lines. Estragole and pulegone, two of the major compounds of *Agastache* sp. EOs, also displayed cytotoxic effects: estragole on SGC-7901 [15] and MCF-7 [17] tumour cell lines and pulegone on the SGC-7901 cancer line [15]. However, the antitumour effect against other cell lines, including colon, liver and triple-negative breast cancer, has not been investigated.

Several *Agastache* sp. have been successfully acclimatised and introduced in culture in Romania [18], raising our interest for their phytochemical and pharmacological investigation. Thus, the aim of our study was to evaluate the chemical composition through gas chromatography-mass spectrometry (GC–MS) analysis, one of the most used methods for the characterisation of essential oils, and to investigate the antibacterial and cytotoxic activities of EOs obtained from the aerial parts of different Romanian cultivated *Agastache* sp.: *A. foeniculum* (AF) and its cultivar “Aromat de Buzău” (AFB), *A. rugosa* (AR) and its cultivar ‘After Eight’ (ARA8) and *A. mexicana* (AM). The cytotoxic effect was tested against a panel of cancer cell lines, representative for triple-negative breast (MDA-MB-231), colon (HCT116), lung (A549) and liver (HEPG2) cancers. This is the first report on the cytotoxic effect of *Agastache* sp. EOs on MDA-MB-231, HCT116 and HEPG2 tumour cell lines. Moreover, AFB is a new Romanian cultivar, with only one study available describing its chemical composition and some pharmacological properties [16], and ARA8 is a cultivar [18] for which studies regarding its chemical composition or therapeutic effects are lacking.

## 2. Results

The EOs were obtained by hydrodistillation with different yields (Table 1); their chemical composition was evaluated using GC–MS analysis, and the in vitro antibacterial and cytotoxic properties were investigated.

### 2.1. GC–MS Profiling

A total of 34 compounds were identified through GC–MS profiling in AF EO, representing 99.63% of the total constituents (Table 2). Menthone was the major compound of the EO (31.58%), followed by estragole (21.80%) and pulegone (21.44%). Limonene (8.69%) and caryophyllene (5.03%) were also identified in significant amounts. Oxygenated monoterpenes represented 54.51% of the AF EO composition, followed by phenylpropanoids (22.39%) and sesquiterpene hydrocarbons (9.23%).

For the AFB EO, 27 compounds were identified (Table 3). The major volatile compound was estragole (63.37%), a phenylpropanoid, followed by limonene (6.29%) and caryophyllene (10.44%). Phenylpropanoids were the predominant class of compounds (69.79%), followed by sesquiterpene hydrocarbons (19.19%) and monoterpene hydrocarbons (6.29%).

The GC–MS analysis of the AM EO led to the identification of 29 different compounds (Table 4), representing 98.46% of the total volatile oil components. The findings revealed that estragole (41.66%) was the main compound, followed by two monoterpenes, pulegone (15.57%) and isomenthone (14.93%). Sesquiterpenes such as caryophyllene, spathulenol and τ-muurolol were also identified.

For AR EO, 29 compounds were identified based on their mass spectra (Table 5). The volatile profile of AR EO was dominated by oxygenated monoterpenes (68.85%) and sesquiterpene hydrocarbons (9.05%). The major components were menthone (39.60%) and pulegone (24.72%). Limonene, estragole and caryophyllene were also found in relatively large quantities.

The analysis of ARA8 EO led to the identification of 28 volatile compounds (Table 6), accounting for 98.41% of the total EO constituents. Menthone (39.76%) and pulegone (27.06%) were found as major constituents; limonene (6.98%), estragole (8.27%) and caryophyllene (3.83%) were also determined in important amounts. The dominant constituents of ARA8 EO were the oxygenated monoterpenes (68.00%), followed by phenylpropanoids (8.92%) and sesquiterpene hydrocarbons (7.49%).

### 2.2. In Vitro Antibacterial Activity of Agastache Species Essential Oils

The results of the antibacterial assay are displayed in Table 7. The following standard strains were tested: *Escherichia coli* ATCC 25922, *Salmonella enteritidis* ATCC 13076, *Staphylococcus aureus* ATCC 6538P, *Listeria monocytogenes* ATCC 19114. AR EO was most active against *E. coli* (8.91 ± 3.27 μL/mL) and *S. aureus* (10.80 ± 0.00 μL/mL) and less active against *S. enteritidis* (22.6 ± 0.00 μL/mL) and *L. monocytogenes* (18.72 ± 6.86 μL/mL). The same pattern was observed for all the tested EOs (AF, AM, ARA8). 

*E. coli* was most susceptible to the inhibitory activity of AR EO, while *S. enteritidis* was most susceptible to ARA8 EO. *S. aureus* was most susceptible to AM EO. 

### 2.3. In Vitro Cytotoxic Activity of Agastache Species Essential Oils

**Figure 1:**
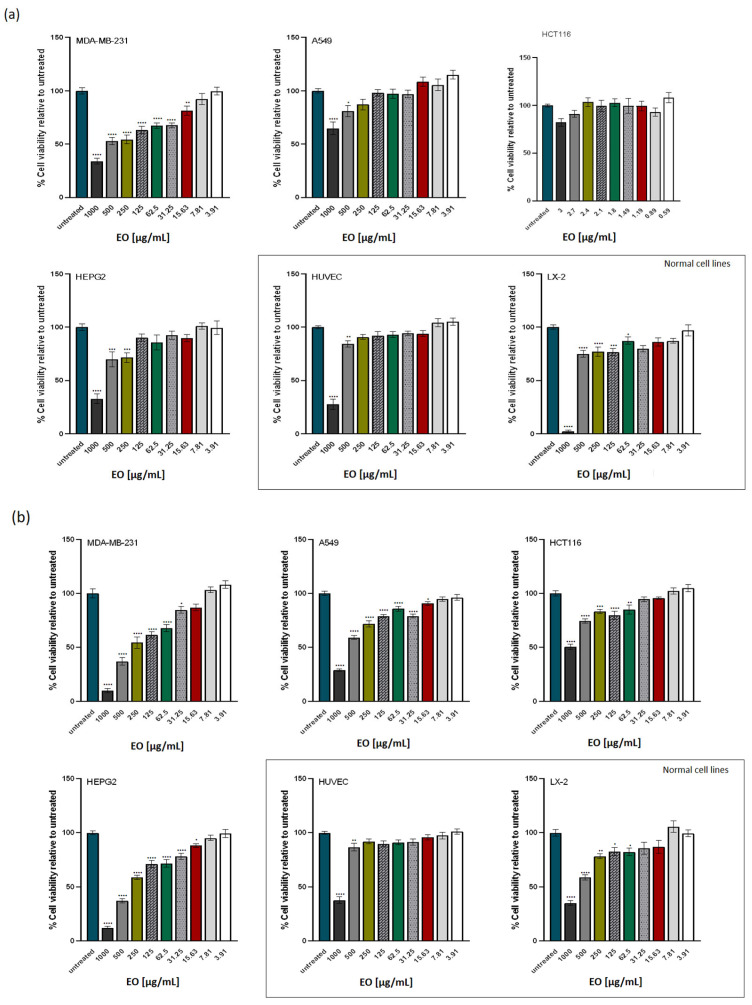
The effect of essential oils on cell viability: (**a**) *A. rugosa* (AR) EO, (**b**) *A. foeniculum* ”Aromat de Buzău” (AFB) EO. The panel of cell lines includes the triple-negative breast (MDA-MB-231), lung (A549), colon (HCT116) and liver (HEPG2) cancer cell lines and normal cell lines represented by the HUVEC (human umbilical endothelial cell) line and LX-2 (hepatic stellate cells). Cells were exposed to the indicated concentrations of EOs for 24 h. Untreated cells (blank) were exposed only to culture medium, while the positive control was represented by cells treated with 100% DMSO. Cell viability was expressed relative to the negative control (considered 100%) and represented as the mean of 3 determinations. Data were analyzed using one-way ANOVA followed by Dunnett’s multiple comparison test. Asterisks *, **, *** and **** indicate significant differences at *p* < 0.05, *p* < 0.01, *p* < 0.001 and *p* < 0.0001, respectively, relative to the corresponding control.

The findings of the MTT assay are displayed in Figure 1. AR EO and AFB EO were used to investigate the in vitro cytotoxic effect, as they displayed the highest concentration of menthone (39.60%) and estragole (63.27%), respectively. In 96-well plates, cells were cultured and treated with full media containing serial dilutions of EO (3.91–1000 µg/mL). Every plate contained a blank control (full media only) and a positive control (100% DMSO). 

The results of the study showed that AR EO had a significant cytotoxic effect on MDA-MB-231 and HEPG2 tumour cell lines, reducing cell viability below 50% at a concentration of 1 mg/mL and a 24 h exposure (Figure 1a). In contrast, AR EO had no significant cytotoxic effect on lung and colon tumour cell lines, cell viability percentage being above 50% for all tested concentrations. No important decrease in cell viability was observed for HUVEC and LX-2 normal cell lines at concentrations below 1 mg/mL.

AFB EO showed superior in vitro cytotoxic activity compared to AR EO on all investigated tumour lines (Figure 1b). AFB EO reduced cell viability below 50% for MDA-MB-231, HEPG2 and A549 tumour cell lines with statistically significant differences relative to the negative control. The most promising effect was on the MDA-MB-231 cancer cell line (IC_50_ = 203.70 ± 0.24 μg/mL), with a well-represented dose–effect relationship. No marked decrease in cell viability was observed for normal cell lines, indicating selectivity over tumour cells.

## 3. Discussion

In the present study, we investigated the chemical composition, as well as the antibacterial and cytotoxic effects of different *Agastache* sp. aerial parts’ EOs. The volatile profiles were evaluated using the GC–MS analysis. The results showed differences in the phytochemical profile depending on species. Menthone, a monoterpene ketone, was the major compound in the EOs of AF, AR and ARA8. In contrast, the major constituent of AFB and AM EOs was estragole, a phenylpropanoid derivative. In AR and ARA8 EOs, estragole was found in a lower concentration (between 4.97% and 8.95%). Moreover, pulegone was found in high amounts in AR and ARA8 EOs (between 11.05 and 27.06%), while AFB EO contained only 0.33–0.46% pulegone. Thus, we identified several categories regarding the chemical composition of *Agastache* sp. EOs, considering the first two major compounds: menthone + estragole (AF), menthone + pulegone (AR, ARA8), estragole + limonene (AFB), estragole + pulegone (AM). Considering the variation in the chemical composition of the essential oils, the following compounds could be considered as markers for the species cultivated in Romania: menthone for AR, ARA8 and AF EOs and estragole for AFB and AM EOs.

Compared to our results, most studies that evaluated the composition of AF EO showed that estragole was the major volatile compound [19,20,21,22,23]. Regarding AR, some studies suggested that estragole was the main component of the EO [24,25,26], while others found methyl eugenol [27], pulegone, menthone [7] or limonene [28] as major compounds. Considering the variability of the chemical composition of the EOs within the same species, several chemotypes have been identified for AR. According to Chae et al. [29], there are five main chemotypes: menthone, menthone + pulegone, methyl eugenol, methyl eugenol + limonene and estragole. Furthermore, two studies pointed out that estragole was the major compound in AM EO [30,31], in accordance with the results of our experiment. 

We identified only one study referring to the chemical composition of EO from AFB, a new Romanian cultivar. Thus, our results are consistent with those reported by Bălănescu et al. [16], who found estragole as the major compound (94.89%), followed by limonene (2.91%) and caryophyllene (0.74%). Other compounds were represented by eugenol, methyl isoeugenol, methyl eugenol and germacrene D, compounds also identified in our study, except for methyl eugenol. In our study, 48 different compounds were identified in AFB EO, of which 7 were also reported by Bălănescu et al. [16], the rest of the compounds being reported for the first time. Regarding ARA8 EO, this is the first study to investigate its chemical composition, identifying menthone as the major compound. 

EOs are well known for their antibacterial properties. Our study showed that the bacterial strains most sensitive to the action of *Agastache* sp. EOs were *E. coli* ATCC 25922 and *S. aureus* ATCC 6538P, *E. coli* being the most sensitive to the action of AR EO (MIC: 8.91 ± 3.27 µL/mL) and *S. aureus* being the most inhibited by AM EO (MIC: 5.14 ± 0.00 µL/mL). In the literature, there are few reports regarding the antibacterial activity of *Agastache* sp. EOs. This is the first study that investigates the antibacterial potential of the EOs from AM, ARA8 and AFB. 

Regarding the antibacterial activity of AR, our results are in accordance with those obtained by Haiyan et al. [7], who found that the EO extracted from AR flowers and leaves showed a moderate to high level of antibacterial activity. The EO from flowers had a significant in vitro inhibitory effect against *S. aureus* (MIC: 21 µg/mL) and *E. coli* (MIC: 21 µg/mL), but the EO from leaves had a higher inhibitory effect against *E. coli* (MIC: 9.4 µg/mL). Another study validated the EO’s antibacterial activity against *S. aureus* and *E. coli*, while also reporting antimicrobial activity against two other Gram-negative bacteria (*Salmonella enteritidis* and *Pseudomonas aeruginosa*) [32]. Compared to our results, other researchers obtained superior inhibitory activity for AF EO on bacterial strains of *L. monocytogenes* (MIC: 1.25 µL/mL) and *Salmonella* sp. (MIC: 1.25 µL/mL) and, in addition, found antibacterial action on other Gram-positive (*Curtobacterium flaccumfaciens*, *Bacillus subtilis*) and Gram-negative (*Proteus vulgaris*, *Klebsiella pneumoniae*) bacteria, with MICs ranging from 0.157 to 2.50 µL/mL [33]. 

Studies have also shown antibacterial properties for the major compounds identified in *Agastache* sp. EOs. Batista et al. found that estragole had antibacterial activity against *S. aures* and *E. coli*, as well as a synergistic effect in combination with gentamicin [34]. Moreover, menthone displayed an antibacterial effect against MRSA, with a minimal inhibitory concentration and minimal bactericidal concentration of 3.54 and 7.08 µg/mL, respectively [35].

Another aim of our study was to investigate the in vitro cytotoxic effect of AFB and AR EOs using the MTT assay. The most promising cytotoxic effect was recorded on MDA-MB-231 and HEPG2 cell lines for both EOs. This is the first study to evaluate the in vitro antiproliferative effect of AR and AFB EOs on MDA-MB-231 and HEPG2 cell lines. Other studies demonstrated that AR and AFB EOs were cytotoxic against MCF-7 hormonal-dependent breast cancer cell line [11,16], while our study showed antiproliferative effects on a non-hormonal cancer cell line, the MDA-MB-231 triple-negative breast cancer cell line. MDA-MB-231 is a highly aggressive, invasive and poorly differentiated cell line, lacking the expression of estrogen and progesterone receptors and showing overexpression of HER2 receptors [36]. Hormonal therapy or targeted therapy cannot be used in triple-negative breast cancer [36]; thus, new therapeutic agents are needed for the treatment of this aggressive pathology.

Few studies have investigated the antiproliferative potential of *Agastache* sp. EOs, and, therefore, information regarding their mechanism of action is limited. However, there are studies that have demonstrated the antitumour effect and mechanism of action of estragole and pulegone, two of the primary constituents of *Agastache* sp. EOs. According to Lashkari et al. (2020) [17], estragole was cytotoxic against the MCF-7 breast cancer line, inducing apoptotic morphological changes, including nuclear fragmentation, chromatin condensation and destruction of cell membrane integrity. Furthermore, estragole and pulegone had antiproliferative effects on the SGC-7901 stomach cancer line at concentrations ranging from 12.5 to 100 µg/mL [15].

Other EOs containing estragole as the major compound also displayed antitumour activity against different cell lines. Thus, *Ocimum basilicum L.* (basil) EO, containing 75.45% estragole, showed in vitro antiproliferative activity against human promyelocytic leukemia cell lines (HL-60 and NB4) and in vivo antitumour activity against Ehrlich ascites carcinoma cells (EACCs) transplanted intraperitoneally in mice [37]. Moreover, another study pointed out that basil EO was cytotoxic against Hep3B (IC_50_ 56.23 ± 1.32 µg/mL) and MCF-7 (80.35 ± 1.17 µg/mL) cell lines [38].

Some studies have shown that high doses of estragole (600 mg/kg body weight) can cause liver cancer in rodents, leading some regulatory agencies to classify it as a possible human carcinogen [39]. However, our study revealed that *Agastache* sp. EOs containing estragole did not display significant cytotoxic activity in vitro against HUVEC and LX-2 normal cell lines, indicating selectivity over tumour cells. 

In our study, AFB EO showed superior cytotoxic activity compared to AR EO on all investigated tumour lines. The differences in the chemical composition could explain the variability of the cytotoxic effect. AFB EO contains 63.27% estragole, a phenylpropanoid derivative that demonstrated antiproliferative effects in vitro on MCF-7 [17] and SGC-7901 [15] tumour cell lines. On the other hand, menthone is the major compound (39.60%) of AR EO, followed by pulegone (24.72%) and limonene (8.36%), with a much lower concentration of estragole (4.97%). However, the chemical composition of the EOs varies significantly. Consequently, the cytotoxic activity may be influenced by the synergistic effect of the active compounds. Further studies are needed to explain the mechanism of action involved in the manifestation of the cytotoxic effect, including morphological analysis and clonogenic cell survival assay.

## 4. Materials and Methods

### 4.1. Plant Material

The plant material, *Agastache rugosa* (Fisch. & C.A.Mey.) Kuntze, *Agastache rugosa* ‘After Eight’, *Agastache foeniculum* (Pursh) Kuntze, *Agastache foeniculum* “Aromat de Buzău”, *Agastache mexicana* (Kunth) Lint & Epling aerial parts were collected in July 2022 during the flowering phase (principal growth stage 6, secondary growth stage 65) [18], from the experimental field of the University of Agricultural Sciences and Veterinary Medicine, Cluj-Napoca (46°45′33.1″ N 23°34′27.7″ E). A voucher specimen was deposited at the Pharmacognosy Department, Faculty of Pharmacy, “Iuliu Hațieganu” University of Medicine and Pharmacy, Cluj-Napoca, Romania: voucher number 137 (AR), 138 (ARA8), 139 (AF), 140 (AFB), 141 (AM). The plant material was air dried at room temperature and ground using an electric grinder.

### 4.2. Essential Oil Obtaining Procedure

The essential oils were obtained from dried aerial parts by hydrodistillation, using a Neo-Clevenger type apparatus. Then, 100 g powdered plant material was boiled in 1 L water, for 3 h [7,40].

### 4.3. GC–MS Profiling

A GC–MS Shimadzu QP 2010 PLUS Mass Spectrometer coupled with Gas Chromatograph (Shimadzu equipped with an AOC-20i+s injector, and a ZB-5MS Plus capillary column (30 m × 0.25 mm, 0.25 µm film thickness, Phenomenex) was used to perform the analysis of the volatile compounds. The MS transfer line and injector temperatures were adjusted to 250 °C. The oven was programmed at 60 °C for 1 min; then, it was set to rise from 60 °C to 120 °C at a rate of 30 °C per minute. It was then programmed to hold this temperature for 5 min, then to increase from 120 °C to 250 °C at a rate of 5 °C per minute, and from 250 °C to 300 °C at a rate of 20 °C per minute. Helium (99.99990% from Linde Hungary) was used as the carrier gas, flowing at a steady 0.8 mL/min. One µL of injection was made at 250 °C in split mode (20:1). The acquisition range (*m*/*z*) of the detector was adjusted at 35 to 800 in scan mode, with the electron impact mode (EI, 70 eV) selected, at an acquisition rate of 500 ms [41]. 

The resulting compounds were identified by comparing their mass spectra to the NIST (NIST 27, 147 libraries), WILEY library database (>90% match), and by calculating their retention indices (RIs) in relation to n-alkanes (C6–C20). Each volatile compound’s relative percentage was calculated as a fraction of its integrated ion area relative to the 100% overall area of the ion chromatogram.

### 4.4. In Vitro Antibacterial Activity

#### 4.4.1. Preparation of Microbial Strains

The antibacterial activity was tested against *Staphylococcus aureus* ATCC 6538P, *Escherichia coli* ATCC 25922, *Salmonella enteritidis* ATCC 13076 and *Listeria monocytogenes* ATCC 19114. Every KWIK-STIK lyophilised microorganism pellet was acquired from Microbiologics, 200 Cooper Avenue North St Cloud, Mn 56303. KWIK-STIKs are widely used for all types of testing of microbiological control.

For twenty-four hours, each strain was cultured in a test tube with 10 mL of sterile nutritional broth (Oxoid Ltd., Basingstoke, Hampshire, UK) at 37 °C. A loopful of the inoculum was transferred onto selective media, which included Palcam agar (Oxoid Ltd., Basingstoke, Hampshire, UK) for *L. monocytogenes*, Baird-Parker agar base supplemented with Egg Yolk Tellurite Emulsion for *S. aureus*, XLD agar for *S. enteritidis* and TBX agar for *E. coli* [42]. The plates were incubated at 37 °C for 24 h. Sterile saline solution (8.5 g/L) was used to transfer multiple colonies of standard cultures that were grown on Mueller–Hinton agar at 37 °C for 24 h. The colonies were adjusted to meet the turbidity of the McFarland 0.5 standard (1.5 × 108 CFU/mL) (McFARLAND). Optical microscopy was used to confirm the bacterial morphology [43].

#### 4.4.2. Determination of the Minimum Inhibitory Concentration (MIC)

A microtiter-plate-based antibacterial test with resazurin was used to determine the MIC. Stock solutions of the EOs were created by mixing 50% ethanol and Tween 80 (8:1) [41]. The first well of a 96-well microtiter plate contained 100 µL of sterile nutrition broth and 100 µL of the sample. To perform serial 11-fold dilutions, 100 µL was moved from one well to another, and 100 µL from the last well in the row was eliminated. Ten µL of inoculum (1.5 × 108 CFU/mL) was added to each well. Gentamicin (0.04 mg/mL in saline solution) was used as a positive control, whereas the negative control was a combination of saline solution, 50% ethanol, and Tween80 (1:8:1). The microplates were incubated at 37 °C for 20 to 22 h. After adding 20 µL of resazurin aqueous solution (0.2 mg/mL) to each well, the microplates were incubated for two hours at 37 °C. The minimum inhibitory concentration (MIC) was the concentration that prevented the blue colour from turning pink. Three replicates were performed for each sample [44].

#### 4.4.3. Determination of the Minimum Bactericidal Concentration (MBC)

The MBC was evaluated by plating 10 μL of aliquot on the Mueller–Hinton medium (Oxoid Ltd., Basingstoke, Hampshire, UK) from the last four wells that showed inhibitory activity against the growth of bacteria during MIC testing. After incubation for 24 h at 37 °C, MBC was determined as the lowest concentration that inhibited bacterial growth. The experiment was performed in triplicate [41,45].

### 4.5. In Vitro Cytotoxic Activity

#### 4.5.1. Cell Lines 

A panel consisting of 4 cancer cell lines and 2 normal cell lines was chosen for in vitro testing of essential oils: triple-negative breast cancer (MDA-MB-231), lung cancer (A549), liver cancer (HEPG2), colon cancer (HCT116). The HUVEC endothelial cell line and LX-2 hepatic stellate cell line was chosen as normal cell lines. All cell lines were maintained in a humidified chamber at 37 °C and 5% CO_2_. All reagents required for cell culture were purchased from Gibco (Dublin, Ireland). Cell lines included in this study were purchased from American Type Culture Collection (ATCC) and provided by the laboratory biobank [46].

#### 4.5.2. Essential Oil Stock Solution Preparation

The essential oils were dehydrated over anhydrous sodium sulfate and stored at −80 °C until the treatment was applied to the cells. The stock solution was prepared at a concentration of 1 mg/mL by dissolving the required amount of volatile oil in the culture medium for each cell line.

#### 4.5.3. MTT Assay

The cytotoxic effect was studied using the MTT test. Cells were cultivated at a density of 1 × 10^4^ cells/well in a 96-well flat-bottom plate, with three duplicates for each treatment. After 24 h of incubation, cells were treated with varying doses of EO (3.91–1000 µg/mL) for 24 h. To remove extract residues that may interfere with the MTT assay, a wash step with phosphate-buffered saline 1× (PBS) was performed. Cell viability was then measured after 5 h of incubation with 100 μL 1 mg/mL MTT. After removing the solution, 150 μL of DMSO was added to dissolve the crystals. The absorbance of viable cells was measured at λ = 570 nm using a SPARK10M multiplate reader (Tecan, Männedorf, Switzerland). Each plate contained a blank control (medium alone) and a positive control (DMSO 100%) [46].

#### 4.5.4. Statistical Analysis

The program Graphpad Prism (version 8) (GraphPad Software, San Diego, CA, USA) was used to conduct the statistical analysis. Cell viability was reported as mean ± SEM and expressed as a percentage of the control group (set at 100%). One-way ANOVA was used to evaluate the data, and then, Dunnett’s multiple-comparison test was performed.

## 5. Conclusions

This study reported a comparative characterisation of the chemical composition of the EOs obtained from different *Agastache* sp. cultivated in Romania. The GC–MS profile of the EOs revealed differences in terms of their major classes of compounds depending on species. AR, ARA8 and AF EOs were dominated by oxygenated monoterpenes, whilst AM and AFB EOs were dominated by phenylpropanoids. Menthone was identified as the most abundant compound in AR, ARA8 and AF, whilst estragole was identified as the major compound in AM and AFB. The evaluation of the antiproliferative activity showed that AR and AFB EOs display significant cytotoxic activity against the MDA-MB-231 and HEPG2 tumour cell lines. Regarding the antibacterial activity, the tested EOs were most active against *E. coli* and *S. aureus*.

This study indicated that the EOs isolated from different Romanian cultivated *Agastache* sp. display important phytochemical diversity, showing in vitro antibacterial and cytotoxic potential against different tumour cell lines. These results provide valuable preliminary data regarding the therapeutic potential of *Agastache* sp. EOs, which warrants further investigation of the mechanism of action involved in the manifestation of the antibacterial and cytotoxic properties and, also, the investigation of these properties in vivo using animal models.

## Figures and Tables

**Table 1:** *Agastache* sp. EOs yields.

Species	EO Yield (mL/100 g)
AF	1.74 ± 0.11
AFB	1.76 ± 0.10
AM	1.79 ± 0.07
AR	1.91 ± 0.08
ARA8	1.71 ± 0.05

AF: *Agastache foeniculum*; AFB: *Agastache foeniculum* “Aromat de Buzău”; AM: *Agastache mexicana*; AR: *Agastache rugosa*; ARA8: *Agastache rugosa* ‘After Eight’; EO: essential oil; sp: species.

**Table 2:** Volatile profile of AF EO (expressed as Area %).

No.	Compound	RI	Area (%)
1.	Limonene (MH)	1026	8.69
2.	Octen-1-ol, acetate (O)	1082	0.30
3.	Menthone (OM)	1151	31.58
4.	*trans*-Isopulegone (OM)	1158	0.65
5.	Estragole (PP)	1178	21.80
6.	Pulegone (OM)	1245	21.44
7.	Piperitone (OM)	1253	0.35
8.	Isopiperitenone (OM)	1281	0.13
9.	2,4-Thujadiene (O)	1124	0.08
10.	1-Cyclohexanone, 2-methyl-2-(3-methyl-2-oxobutyl) (O)	1143	0.06
11.	Carveol acetate (OM)	1348	0.08
12.	ɣ-Elemene (SH)	1435	0.51
13.	Eucarvone (OM)	1248	0.28
14.	Eugenol (PP)	1352	0.13
15.	ß-Bourbonene (SH)	1378	0.45
16.	ß-Elemene (SH)	1392	0.16
17.	Methylchavibetol (PP)	1305	0.46
18.	Caryophyllene (SH)	1447	5.03
19.	ß-Cubebene (SH)	1390	0.51
20.	α-Amorphene (SH)	1490	0.19
21.	ɣ-Cadinene (SH)	1512	0.21
22.	α-Caryophyllene (SH)	1457	0.18
23.	Germacrene D—isomer (SH)	1495	0.08
24.	Ylangene (SH)	1476	0.34
25.	Germacrene D—isomer (SH)	1495	0.17
26.	ɣ-Elemene (SH)	1435	0.43
27.	α-Farnesene (SH)	1503	0.26
28.	(+)-δ-Cadinene (SH)	1523	0.71
29.	(−)-Spathulenol (OS)	1578	1.42
30.	Caryophyllene oxide (OS)	1581	1.02
31.	Spathulenol (OS)	1619	0.17
32.	α-Cadinol (OS)	1653	0.47
33.	τ-Muurolol (OS)	1638	0.88
34.	Phytol (O)	2085	0.41
	Total identified		34 (99.63%)
	PP		3 (22.39%)
	MH		1 (8.69%)
	OM		7 (54.51%)
	SH		14 (9.23%)
	OS		5 (3.96%)
	O		4 (0.85%)

AF—*Agastache foeniculum*, PP—phenylpropanoids, MH-monoterpene hydrocarbons, OM—oxygenated monoterpenes, SH—sesquiterpene hydrocarbons, OS—oxygenated sesquiterpenes, O—other; RI—retention index.

**Table 3:** Volatile profile of AFB EO (expressed as Area %).

No.	Compound	RI	Area (%)
1.	Limonene (MH)	1026	6.29
2.	Estragole (PP)	1178	63.27
3.	Pulegone (OM)	1245	0.46
4.	Anethole (PP)	1287	0.09
5.	γ-Elemene (SH)	1435	1.11
6.	Eugenol (PP)	1352	0.55
7.	Chavibetol (PP)	1371	1.54
8.	β-Bourbonene (SH)	1378	0.17
9.	(−)-β-Elemene (SH)	1392	0.12
10.	Methyl isoeugenol (PP)	1397	4.34
11.	Caryophyllene (SH)	1457	10.44
12.	β-Cuvebene (SH)	1532	1.78
13.	(E)-β-Farnesene (SH)	1462	0.71
14.	γ-Cadinene (isomer) (SH)	1512	0.83
15.	α-Caryophyllene (SH)	1457	0.47
16.	(+)-Epi-bicyclosesquiphellandrene (SH)	1486	0.24
17.	Ylangene (SH)	1476	0.75
18.	Germacrene D (isomer) (SH)	1495	0.75
19.	γ -Elemene (SH)	1435	0.66
20.	γ-Cadinene (isomer) (SH)	1512	0.49
21.	(+)-δ-Cadinene (SH)	1523	0.67
22.	(−)-Spathulenol (OS)	1578	0.95
23.	Caryophyllene oxide (OS)	1581	0.49
24.	α-Cadinol (OS)	1653	0.82
25.	Hexahydrofarnesyl acetone (OS)	1816	0.16
26.	Pentadecanoic acid (O)	1852	0.31
27.	Phytol (O)	2085	1.54
	Total		27 (100%)
	PP		5 (69.79%)
	MH		1 (6.29%)
	OM		1 (0.46%)
	SH		14 (19.19%)
	OS		4 (2.42%)
	O		2 (1.85%)

AFB—*Agastache foeniculum* “Aromat de Buzău”, PP—phenylpropanoids, MH-monoterpene hydrocarbons, OM—oxygenated monoterpenes, SH—sesquiterpene hydrocarbons, OS—oxygenated sesquiterpenes, O—other; RI—retention index.

**Table 4:** Volatile profile of AM EO (expressed as Area %).

No.	Compound	RI	Area (%)
1.	1-Octen-3-ol (O)	976	0.92
2.	Limonene (MH)	1026	6.94
3.	Octen-1-ol, acetate (O)	1082	0.62
4.	(+)-Isomenthone (OM)	1166	14.93
5.	Estragole (PP)	1178	41.66
6.	(−)-2,3-Pinanediol (OM)	1311	0.37
7.	Pulegone (OM)	1245	15.57
8.	Piperitone (OM)	1253	0.21
9.	Elixene (isomer) (SH)	1364	0.61
10.	Eucarvone (OM)	1248	0.37
11.	Eugenol (PP)	1352	0.29
12.	β-Bourbonene (SH)	1378	0.31
13.	(−)-β-Elemene (SH)	1392	0.20
14.	Methylchavibetol (PP)	1305	0.14
15.	Caryophyllene (SH)	1457	6.86
16.	β-Cubebene (SH)	1532	0.57
17.	α-Amorphene (SH)	1490	0.19
18.	γ-Cadinene (isomer) (SH)	1512	0.24
19.	α-Caryophyllene (SH)	1457	0.28
20.	Germacrene D (SH)	1495	0.18
21.	Elixene (isomer) (SH)	1364	0.50
22.	α-Farnesene (SH)	1503	0.28
23.	γ-Cadinene (isomer) (SH)	1512	0.08
24.	(+)-δ-Cadinene (SH)	1523	0.64
25.	Spathulenol (OS)	1619	2.03
26.	Caryophyllene oxide (OS)	1581	1.37
27.	α-Cadinol (OS)	1653	0.37
28.	τ-muurolol (OS)	1638	1.03
29.	Phytol (O)	2085	0.70
	Total identified		29 (98.46%)
	PP		3 (42.09%)
	MH		1 (6.94%)
	OM		5 (31.45%)
	SH		13 (10.94%)
	OS		4 (4.8%)
	O		3 (2.24%)

AM—*Agastache mexicana*, PP—phenylpropanoids, MH-monoterpene hydrocarbons, OM—oxygenated monoterpenes, SH—sesquiterpene hydrocarbons, OS—oxygenated sesquiterpenes, O—other; RI—retention index.

**Table 5:** Volatile profile of AR EO (expressed as Area %).

No.	Compound	RI	Area (%)
1.	Limonene (MH)	1026	8.36
2.	Menthone (OM)	1150	39.60
3.	Estragole (PP)	1195	4.97
4.	Pulegone (OM)	1245	24.72
5.	Piperitone (OM)	1253	0.67
6.	Lavandulyl acetate (OM)	1293	0.63
7.	(−)-Myrtenyl acetate (OM)	1238	0.19
8.	1-Cyclohexanone, 2-methyl-2-(3-methyl-2-oxobutyl) (O)	1143	0.20
9.	Carvyl acetate (OM)	1335	0.13
10.	γ-Elemene (SH)	1435	0.13
11.	Elixene (SH)	1364	0.73
12.	Eucarvone (OM)	1245	0.62
13.	(Z)-Geranyl acetate (OM)	1357	1.36
14.	(E)-Geranyl acetate (OM)	1383	0.93
15.	β-Bourbonene (SH)	1378	0.56
16.	(−)-β-Elemene (SH)	1392	0.14
17.	Caryophyllene (SH)	1447	4.77
18.	β-Cubebene (SH)	1532	0.59
19.	Germacrene D (SH)	13.78	0.18
20.	α-Caryophyllene (SH)	1457	0.16
21.	γ-Elemene (SH)	1435	0.50
22.	α-Farnesene (SH)	1503	0.46
23.	(+)-δ-Cadinene (SH)	1523	0.83
24.	Spathulenol (OS)	1619	2.22
25.	Caryophyllene oxide (OS)	1581	1.37
26.	α-Cadinol (OS)	1653	0.58
27.	α-Bisabolol (OS)	1693	0.76
28.	Pentadecanoic acid (O)	1852	0.37
29.	Phytol (O)	2085	0.77
	Total identified		29 (97.5%)
	PP		1 (4.97%)
	MH		1 (8.36%)
	OM		9 (68.85%)
	SH		11 (9.05%)
	OS		4 (4.93%)
	O		3 (1.34%)

AR—*Agastache rugosa*, PP—phenylpropanoids, MH-monoterpene hydrocarbons, OM—oxygenated monoterpenes, SH—sesquiterpene hydrocarbons, OS—oxygenated sesquiterpenes, O—other; RI—retention index.

**Table 6:** Volatile profile of ARA8 EO (expressed as Area %).

No.	Compound	RI	Area (%)
1.	1-Octyn-3-ol, 4-ethyl- (O)	982	1.66
2.	Limonene (MH)	1026	6.98
3.	Menthone (OM)	1150	39.76
4.	Estragole (PP)	1178	8.27
5.	Pulegone (OM)	1245	27.06
6.	Piperitone (OM)	1253	1.04
7.	1-Cyclohexanone, 2-methyl-2-(3-methyl-2-oxobutyl) (O)	1143	0.40
8.	Carveol acetate (OM)	1348	0.14
9.	Elixene (SH)	1364	0.48
10.	Eugenol (PP)	1352	0.54
11.	β-Bourbonene (SH)	1378	0.41
12.	(−)-β-Elemene (SH)	1392	0.10
13.	Methylchavibetol (PP)	1305	0.11
14.	Caryophyllene (SH)	1447	3.83
15.	β-Cubebene (SH)	1532	0.56
16.	Germacrene D (SH)	1495	0.18
17.	γ-Cadinene (SH)	1512	0.20
18.	α-Caryophyllene (SH)	1457	0.12
19.	Ylangene (SH)	14.60	0.28
20.	Germacrene D (SH)	1495	0.17
21.	γ-Elemene (SH)	1435	0.22
22.	α-Farnesene (SH)	1503	0.46
23.	(+)-δ-Cadinene (SH)	15.69	0.48
24.	(−)-Spathulenol (OS)	1619	1.87
25.	Caryophyllene oxide (OS)	1581	1.20
26.	(+)-Spathulenol (OS)	1578	0.26
27.	α-Cadinol (OS)	1653	0.99
28.	Phytol (O)	2085	0.64
	Total identified		28 (98.41%)
	PP		3 (8.92%)
	MH		1 (6.98%)
	OM		4 (68.00%)
	SH		13 (7.49%)
	OS		4 (4.32%)
	O		3 (2.70%)

ARA8—*Agastache rugosa* ‘After Eight’, PP—phenylpropanoids, MH-monoterpene hydrocarbons, OM—oxygenated monoterpenes, SH—sesquiterpene hydrocarbons, OS—oxygenated sesquiterpenes, O—other; RI—retention index.

**Table 7:** Antibacterial activity of *Agastache* species essential oils.

Samples	*Escherichia coli* ATCC 25922		*Salmonella enteritidis* ATCC 13076		*Staphylococcus aureus* ATCC 6538P		*Listeria monocytogenes* ATCC 19114	
	MIC (μL/mL)	MBC (μL/mL)	MIC (μL/mL)	MBC (μL/mL)	MIC (μL/mL)	MBC (μL/mL)	MIC (μL/mL)	MBC (μL/mL)
AR EO	8.91 ± 3.27 ^d^	22.68 ± 0.00 ^b^	22.6 ± 0.00 ^c^	22.68 ± 0.00 ^b^	10.80 ± 0.00 ^c^	18.72 ± 6.86 ^c^	18.72 ± 6.86 ^d^	22.68 ± 0.00 ^b^
ARA8 EO	10.80 ± 0.00 ^c^	22.68 ± 0.00 ^b^	18.72 ± 6.86 ^d^	22.68 ± 0.00 ^b^	8.91 ± 3.27 ^d^	22.68 ± 0.00 ^b^	18.72 ± 6.86 ^d^	22.68 ± 0.00 ^b^
AF EO	18.72 ± 6.86 ^b^	18.72 ± 6.86 ^c^	22.68 ± 0.00 ^c^	22.68 ± 0.00 ^b^	10.80 ± 0.00 ^c^	18.72 ± 6.86 ^c^	22.68 ± 0.00 ^c^	22.68 ± 0.00 ^b^
AFB EO	18.72 ± 6.86 ^b^	22.68 ± 0.00 ^b^	30.99 ±14.39 ^b^	47.62 ± 0.00 ^a^	22.68 ± 0.00 ^b^	47.62 ± 0.00 ^a^	22.68 ± 0.00 ^c^	22.68 ± 0.00 ^b^
AM EO	10.80 ±0.00 ^c^	22.68 ± 0.00 ^b^	22.68 ± 0.00 ^c^	22.68 ± 0.00 ^b^	5.14 ± 0.00 ^e^	10.80 ± 0.00 ^d^	22.68 ± 0.00 ^c^	22.68 ± 0.00 ^b^
Gentamicin (μg/mL)	0.24 ± 0.00	0.24 ± 0.00	0.50 ± 0.73	0.50 ± 0.73	0.05 ± 0.73	0.05 ± 0.73	0.50 ± 0.00	0.50 ± 0.00

Note: Values are expressed as mean of three replicates ± SD. Means with different letters (a,b,c,d,e) within a column indicate significant differences (*p* < 0.05) using Tukey’s Honestly Significant Differences (HSD) test with a confidence interval of 95% or 99%. MIC: minimum inhibitory concentration; MBC: minimum bactericidal concentration; AR: *Agastache rugosa*; ARA8: *Agastache rugosa* ‘After Eight’; AF: *Agastache foeniculum*; AFB: *Agastache foeniculum* “Aromat de Buzău”; AM: *Agastache mexicana.*

## Data Availability

The raw data supporting the conclusions of this article will be made available by the authors on request.

## Abbreviations

*Agastache foeniculum* (AF), *Agastache foeniculum* “Aromat de Buzău” (AFB), *Agastache rugosa* (AR), *Agastache rugosa* ‘After Eight’ (ARA8), *Agastache mexicana* (AM), essential oil(s) (EO(s)), gas chromatography-mass spectrometry (GC–MS), minimum inhibitory concentration (MIC), minimum bactericidal concentration (MB), species (sp.)

## References

[1] Hernández-Abreu O., Castillo-España P., León-Rivera I., Ibarra-Barajas M., Villalobos-Molina R., González-Christen J., Vergara-Galicia J., Estrada-Soto S. (2009). Antihypertensive and Vasorelaxant Effects of Tilianin Isolated from *Agastache mexicana* Are Mediated by NO/CGMP Pathway and Potassium Channel Opening. Biochem. Pharmacol..

[2] Wesołowska A. (2019). Influence of Distillation Time on the Content and Composition of Essential Oils Isolated from Different Parts of *Agastache astromontana* ‘Pink Pop’. J. Essent. Oil Bear. Plants.

[3] Verano J., González-Trujano M.E., Déciga-Campos M., Ventura-Martínez R., Pellicer F. (2013). Ursolic Acid from *Agastache mexicana* Aerial Parts Produces Antinociceptive Activity Involving TRPV1 Receptors, CGMP and a Serotonergic Synergism. Pharmacol. Biochem. Behav..

[4] Najar B., Marchioni I., Ruffoni B., Copetta A., Pistelli L., Pistelli L. (2019). Volatilomic Analysis of Four Edible Flowers from *Agastache* Genus. Molecules.

[5] Yuk H.J., Ryu H.W., Kim D.-S. (2023). Potent Xanthine Oxidase Inhibitory Activity of Constituents of *Agastache rugosa* (Fisch. and C.A.Mey.) Kuntze. Foods.

[6] Nam H.-H., Kim J.S., Lee J., Seo Y.H., Kim H.S., Ryu S.M., Choi G., Moon B.C., Lee A.Y. (2020). Pharmacological Effects of *Agastache rugosa* against Gastritis Using a Network Pharmacology Approach. Biomolecules.

[7] Haiyan G., Lijuan H., Shaoyu L., Chen Z., Ashraf M.A. (2016). Antimicrobial, Antibiofilm and Antitumor Activities of Essential Oil of *Agastache rugosa* from Xinjiang, China. Saudi J. Biol. Sci..

[8] Najafi F., Kavoosi G., Siahbalaei R., Kariminia A. (2022). Anti-Oxidative and Anti-Hyperglycemic Properties of *Agastache foeniculum* Essential Oil and Oily Fraction in Hyperglycemia-Stimulated and Lipopolysaccharide-Stimulated Macrophage Cells: In Vitro and in Silico Studies. J. Ethnopharmacol..

[9] Park C.H., Yeo H.J., Baskar T.B., Park Y.E., Park J.S., Lee S.Y., Park S.U. (2019). In Vitro Antioxidant and Antimicrobial Properties of Flower, Leaf, and Stem Extracts of Korean Mint. Antioxidants.

[10] Moon H., Kim M.J., Son H.J., Kweon H.-J., Kim J.T., Kim Y., Shim J., Suh B.-C., Rhyu M.-R. (2015). Five HTRPA1 Agonists Found in Indigenous Korean Mint, *Agastache rugosa*. PLoS ONE.

[11] Sun J., Sun P., Kang C., Zhang L., Guo L., Kou Y. (2022). Chemical Composition and Biological Activities of Essential Oils from Six *Lamiaceae* Folk Medicinal Plants. Front. Plant Sci..

[12] Hwang J.M., Lee M.-H., Lee J.-H., Lee J.H. (2021). *Agastache rugosa* Extract and Its Bioactive Compound Tilianin Suppress Adipogenesis and Lipogenesis on 3T3-L1 Cells. Appl. Sci..

[13] Lee J.-J., Lee J., Gu M., Han J.-H., Cho W.-K., Ma J. (2017). *Agastache rugosa* Kuntze Extract, Containing the Active Component Rosmarinic Acid, Prevents Atherosclerosis through up-Regulation of the Cyclin-Dependent Kinase Inhibitors P21WAF1/CIP1 and P27KIP1. J. Funct. Foods.

[14] Kim M., Chung W., Kim Y., Lee J., Lee H., Hwang B., Park Y., Hwang S., Kim J. (2001). The Effect of the Oil of *Agastache rugosa* O. Kuntze and Three of Its Components on Human Cancer Cell Lines. J. Essent. Oil Res..

[15] Gong H., Li S., He L., Kasimu R. (2017). Microscopic Identification and in Vitro Activity of *Agastache rugosa* (Fisch. et Mey) from Xinjiang, China. BMC Complement. Altern. Med..

[16] Bălănescu F., Botezatu A.V., Marques F., Busuioc A., Marincaş O., Vînătoru C., Cârâc G., Furdui B., Dinica R.M. (2023). Bridging the Chemical Profile and Biological Activities of a New Variety of *Agastache foeniculum* (Pursh) Kuntze Extracts and Essential Oil. Int. J. Mol. Sci..

[17] Lashkari A., Najafi F., Kavoosi G., Niazi S. (2020). Evaluating the In Vitro Anti-Cancer Potential of Estragole from the Essential Oil of *Agastache foeniculum* [Pursh.] Kuntze. Biocatal. Agric. Biotechnol..

[18] Vârban R., Ona A., Stoie A., Vârban D., Crișan I. (2021). Phenological Assessment for Agronomic Suitability of Some *Agastache* Species Based on Standardized BBCH Scale. Agronomy.

[19] Stefan D.-S., Popescu M., Luntraru C.-M., Suciu A., Belcu M., Ionescu L.-E., Popescu M., Iancu P., Stefan M. (2022). Comparative Study of Useful Compounds Extracted from *Lophanthus anisatus* by Green Extraction. Molecules.

[20] Mahmoodi M., Malekzadeh M., Tava A. (2014). Influence of Drying, Storage and Distillation Times on Essential Oil Yield and Composition of Anise Hyssop [*Agastache foeniculum* (Pursh.) Kuntze]. J. Essent. Oil Res..

[21] Ebadollahi A., Safaralizadeh M., Pourmirza A., Gheibi S. (2010). Toxicity of Essential Oil of *Agastache foeniculum* (Pursh) Kuntze to *Oryzaephilus surinamensis* L. and *Lasioderma serricorne* F. J. Plant Prot. Res..

[22] Mohammadi H., Moradi S., Hazrati S., Aghaee A. (2022). Melatonin Application on Phytochemical Compositions of *Agastache foeniculum* under Water-Deficit Stress. Bot. Sci..

[23] Mallavarapu G., Kulkarni R., Baskaran K., Ramesh S. (2004). The Essentials Oil Composition of Anise Hysop Grown in India. Flavour Fragr. J..

[24] Shutava H.G., Kavalenka N.A., Supichenka N.N., Leontiev N.N., Shutava N.G. (2014). Essential Oils of Lamiaceae with High Content of α-, β-Pinene and Limonene Enantiomers. J. Essent. Oil Bear. Plants.

[25] Yamani H., Mantri N., Morrison P.D., Pang E. (2014). Analysis of the Volatile Organic Compounds from Leaves, Flower Spikes, and Nectar of Australian Grown *Agastache rugosa*. BMC Complement. Altern. Med..

[26] Anand S., Deighton M., Livanos G., Pang E.C.K., Mantri N. (2019). Agastache Honey Has Superior Antifungal Activity in Comparison with Important Commercial Honeys. Sci. Rep..

[27] Li H.Q., Liu Q.Z., Liu Z.L., Du S.S., Deng Z.W. (2013). Chemical Composition and Nematicidal Activity of Essential Oil of *Agastache rugosa* against *Meloidogyne incognita*. Molecules.

[28] Jun H.-J., Chung M.J., Dawson K., Rodriguez R.L., Houng S.-J., Cho S.-Y., Jeun J., Kim J.-Y., Kim K.H., Park K.W. (2010). Nutrigenomic Analysis of Hypolipidemic Effects of *Agastache rugosa* Essential Oils in HepG2 Cells and C57BL/6 Mice. Food Sci. Biotechnol..

[29] Chae Y.-A., Hyun-Choong O. (2005). Variability of the Volatile Composition of *Agastache rugosa* in South Korea. Acta Hortic..

[30] Estrada-Reyes R., Aguirre Hernández E., García-Argáez A., Soto Hernández M., Linares E., Bye R., Heinze G., Martínez-Vázquez M. (2004). Comparative Chemical Composition of *Agastache mexicana* subsp. *mexicana* and *A. mexicana* subsp. *xolocotziana*. Biochem. Syst. Ecol..

[31] Navarrete A., Ávila-Rosas N., Majín-León M., Balderas-López J.L., Alfaro-Romero A., Tavares-Carvalho J.C. (2017). Mechanism of Action of Relaxant Effect of *Agastache mexicana* ssp. *mexicana* Essential Oil in Guinea-Pig Trachea Smooth Muscle. Pharm. Biol..

[32] Kim J. (2008). Phytotoxic and Antimicrobial Activities and Chemical Analysis of Leaf Essential Oil from *Agastache rugosa*. J. Plant Biol..

[33] Ivanov I.G., Vrancheva R.Z., Petkova N.T., Tumbarski Y., Dincheva I.N., Badjakov I.K. (2019). Phytochemical Compounds of Anise Hyssop (*Agastache foeniculum*) and Antibacterial, Antioxidant, and Acetylcholinesterase Inhibitory Properties of Its Essential Oil. J. Appl. Pharm. Sci..

[34] Batista F.L.A., Andrade-Pinheiro J.C., dos Santos A.T.L., Lima J.N.M., Alencar G.G., Siqueira G.M., da Silva A.R.P., de Carvalho N.K.G., Martins A.O.B.P.B., da Costa R.H.S. (2023). Comparative Antimicrobial Potential of *Ocimum basilicum* Essential Oil, Estragole and Estragole/β-Cyclodextrin Complex in an Infection Model on Adult Zebrafish. Carbohydr. Polym. Technol. Appl..

[35] Zhao W., Yang C., Zhang N., Peng Y., Ma Y., Gu K., Liu X., Liu X., Liu X., Liu Y. (2023). Menthone Exerts Its Antimicrobial Activity Against Methicillin Resistant *Staphylococcus aureus* by Affecting Cell Membrane Properties and Lipid Profile. Drug Des. Dev. Ther..

[36] Hero T., Bühler H., Kouam P.N., Priesch-Grzeszowiak B., Lateit T., Adamietz I.A. (2019). The Triple-Negative Breast Cancer Cell Line MDA-MB 231 Is Specifically Inhibited by the Ionophore Salinomycin. Anticancer. Res..

[37] Mahmoud G.I. (2013). Biological effects, antioxidant and anticancer activities of marigold and basil essential oils. J. Med. Plants Res..

[38] Eid A.M., Jaradat N., Shraim N., Hawash M., Issa L., Shakhsher M., Nawahda N., Hanbali A., Barahmeh N., Taha B. (2023). Assessment of Anticancer, Antimicrobial, Antidiabetic, Anti-Obesity and Antioxidant Activity of *Ocimum basilicum* Seeds Essential Oil from Palestine. BMC Complement. Med. Ther..

[39] European Medicines Agency (2015). Public Statement on the Use of Herbal Medicinal Products Containing Estragole (Revision 1). https://www.ema.europa.eu/en/documents/other/public-statement-use-herbal-medicinal-products-containing-estragole-revision-1_en.pdf.

[40] Toma C.-C., Simu G.M., Hanganu D., Olah N., Vata F.M.G., Hammami C., Hammami M. (2010). Chemical composition of the tunisian *Nigella sativa*. Farmacia.

[41] Vârban D., Zăhan M., Crișan I., Pop C.R., Gál E., Ștefan R., Rotar A.M., Muscă A.S., Meseșan Ș.D., Horga V. (2023). Unraveling the Potential of Organic Oregano and Tarragon Essential Oils: Profiling Composition, FT-IR and Bioactivities. Plants.

[42] Bodea I.M., Cătunescu G.M., Pop C.R., Fiț N.I., David A.P., Dudescu M.C., Stănilă A., Rotar A.M., Beteg F.I. (2022). Antimicrobial Properties of Bacterial Cellulose Films Enriched with Bioactive Herbal Extracts Obtained by Microwave-Assisted Extraction. Polymers.

[43] Semeniuc C.A., Pop C.R., Rotar A.M. (2017). Antibacterial Activity and Interactions of Plant Essential Oil Combinations against Gram-Positive and Gram-Negative Bacteria. J. Food Drug Anal..

[44] Vârban D., Zăhan M., Pop C.R., Socaci S., Ștefan R., Crișan I., Bota L.E., Miclea I., Muscă A.S., Deac A.M. (2022). Physicochemical Characterization and Prospecting Biological Activity of Some Authentic Transylvanian Essential Oils: Lavender, Sage and Basil. Metabolites.

[45] Cerezo A.B., Cătunescu G.M., González M.M.-P., Hornedo-Ortega R., Pop C.R., Rusu C.C., Chirilă F., Rotar A.M., Garcia-Parrilla M.C., Troncoso A.M. (2020). Anthocyanins in Blueberries Grown in Hot Climate Exert Strong Antioxidant Activity and May Be Effective against Urinary Tract Bacteria. Antioxidants.

[46] Pralea I.-E., Moldovan R.-C., Țigu A.-B., Petrache A.-M., Hegheș S.-C., Mitoi M., Cogălniceanu G., Iuga C.-A. (2022). Profiling of Polyphenolic Compounds of Leontopodium Alpinum Cass Callus Cultures Using UPLC/IM-HRMS and Screening of In Vitro Effects. Plants.

